# Negative Photoconductivity of Ag-Poly(heptazine imide)

**DOI:** 10.1021/acsomega.5c02134

**Published:** 2025-07-08

**Authors:** Chihiro Miyazaki, Momoka Isobe, Yunosuke Takezawa, Ayane Nakamura, Mai Hattori, Ryosuke Ohnuki, Shinya Yoshioka, Kaname Kanai

**Affiliations:** Department of Physics and Astronomy, Faculty of Science and Technology, 13258 Tokyo University of Science, 2641 Yamazaki, Noda, Chiba 278-8510, Japan

## Abstract

Poly­(heptazine imide) (PHI), a covalent organic framework containing metal ions, is a novel, visible-light-driven photocatalyst. The physical properties of PHI vary depending on the type of metal ion. The photoresponse current of Ag-PHI exhibits negative photoconductivity (NPC), in contrast to PHIs containing many other metals. This is the first PHI material exhibiting NPC, which is extremely rare among organic semiconductors. Ag nanoparticles (Ag-NPs) produced by white-light irradiation of Ag-PHI are essential in the NPC mechanism. Ag^+^ released from the PHI structure aggregates into Ag-NPs under photoirradiation, with the formation of Ag-PHI black. The color of Ag-PHI black is black due to the light absorption of the surface plasmon resonance of the Ag-NPs it contains. The Ag-NPs in Ag-PHI black trap the photogenerated holes, reducing the number of carriers and forming an electron injection barrier at the electrode interface, thereby decreasing the current. Upon discontinuing photoirradiation, the holes trapped in the Ag-NPs were released, and the current increased gradually. The proposed mechanism not only explains the NPC exhibited by Ag-PHI but also provides important insights for developing organic semiconductors that exhibit NPC.

## Graphical abstract

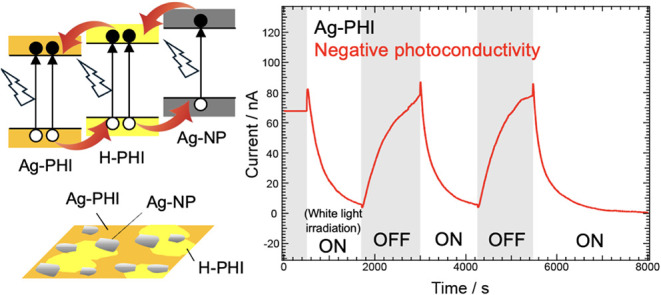

## 1 Introduction

Generally, the electrical conduction of semiconductors follows two mechanisms. In the normal conduction mechanism, application of a bias to an inorganic semiconductor induces conduction because of the drift conduction of the carriers in the semiconductor. In the alternative photoconduction mechanism, excitons are generated when a semiconductor absorbs light. Some of the excitons separate into charge carriers, and due to holes remaining in the valence band and electrons remaining in the conduction band as free carriers, the carrier density increases, thereby improving the electrical conductivity. This photoconductivity is commonly observed in both inorganic and organic semiconductors, is called positive photoconductivity (PPC). However, extremely rare substances exhibit a decrease in the electrical conductivity upon photoirradiation. This unusual phenomenon is known as negative photoconductivity (NPC).^1^ The mechanism of PPC is intuitively understood, whereas there is still no widely applicable model for the mechanism of NPC; therefore, microscopic understanding is a major challenge.

NPC has been observed in a wide range of materials, including Si with Au loading,^2,3^ graphene quantum dots (GQD),^4,5^ reduced graphene oxide with Au nanoparticle loading,^6^ PbTe,^7^ and 2D materials. However, most of these are inorganic materials,^8^ whereas reports of organic materials exhibiting NPC are lacking. NPC has also been reported in organic–inorganic hybrid materials such as graphene, carbon nanotubes,^9^ and halide perovskites. However, reports of NPC in organic semiconductors are limited.^10–12^ Individual models have been proposed to explain the mechanisms in materials exhibiting NPC, including carrier trapping due to deep trap levels inherent in semiconductors,^13^ carrier scattering due to water molecules adsorbed on the semiconductor surface,^4,14^ the trion effect,^15^ photoactivated trap states,^16^ surface plasmon resonance due to metal nanoparticles (NPs),^17^ and intraband scattering.^18^

Although the NPC mechanism has not been fully elucidated, NPC is believed to have wide applicability. For example, for light-detection devices, when a semiconductor exhibiting NPC is illuminated, the current decreases; thus, the power consumption is lower than that of light detection using PPC. NPC can also enable ultrahigh-sensitivity light detection^16,19^ and single-wavelength light detectors by exploiting the strong dependence on the energy of incident light.^20,21^ Various logic gate circuits have been realized by combining the light responses of PPC and NPC using semiconductor homojunctions based on 2D materials and SnO_2_–NPs.^22,23^ Because NPC is generated in GQD and CsPbBr_3_/graphene by the adsorption of water molecules on the semiconductor surface, NPC can prospectively be applied to humidity sensors.^4,5,14^ Application in synaptic devices is also being considered for mimicking the functions of the human eye and brain using organic molecule-nanowire heterojunctions and wide-bandgap oxide-based devices.^24,25^

The authors found that Ag-poly­(heptazine imide) (Ag-PHI), a covalent organic framework (COF), exhibits NPC. This is the first COF to exhibit NPC and the first example of an organic-based semiconductor with NPC. A COF is a porous crystalline organic structure with a framework formed by covalent bonds of light elements such as carbon and nitrogen.^26^ PHI is a COF containing a metal ion. PHIs containing alkali metal ions, such as K-PHI, have been extensively studied.^27–29^ PHI is a multifunctional COF that is both a semiconductor and an ion conductor.^27,30^ PHI materials have attracted attention as new, visible-light-driven photocatalysts.^31,32^ As shown in Figure 1, the structure of Ag-PHI contains Ag^+^ ions within ∼1.3 nm wide pores in the PHI framework. The number of Ag^+^ ions contained in the pores of Ag-PHI has not yet been experimentally confirmed. However, given the large ionic radius of Ag^+^ and recent reports indicating that the number of K^+^ ions contained in the pores of K-PHI is approximately one,^30^ it can be speculated that the number of Ag^+^ ions contained in the pores of Ag-PHI is likely to be no more than one. Upon exposure to white light, the color of Ag-PHI changes irreversibly from yellow to black, unlike the photochromism exhibited by other PHIs, such as K-PHI.^33–35^ This change is caused by Ag-NPs formed by the aggregation of Ag^+^ released from the pores of the PHI framework upon photoirradiation. Broadband optical absorption in the visible-light region due to the surface plasmon resonance of Ag-NPs makes Ag-PHI black. In addition, Ag-NPs can cause NPC. In this study, a model for the NPC mechanism in Ag-PHI is proposed based on several experimental observations. As mentioned above, in some inorganic materials, NPC can be caused by metal NPs acting as carrier traps. On the other hand, in the case of Ag-PHI, it can also be pointed out that NPC may be caused by the carrier injection barrier at the electrode interface formed when Ag-PHI is positively charged, with the metal Ag-NPs trapping holes. This unique mechanism of NPC should provide insights into the mechanisms of NPC in other substances. The knowledge obtained in this study is useful for research on new functions of PHI and new organic substances that exhibit NPC.

**Figure 1:**
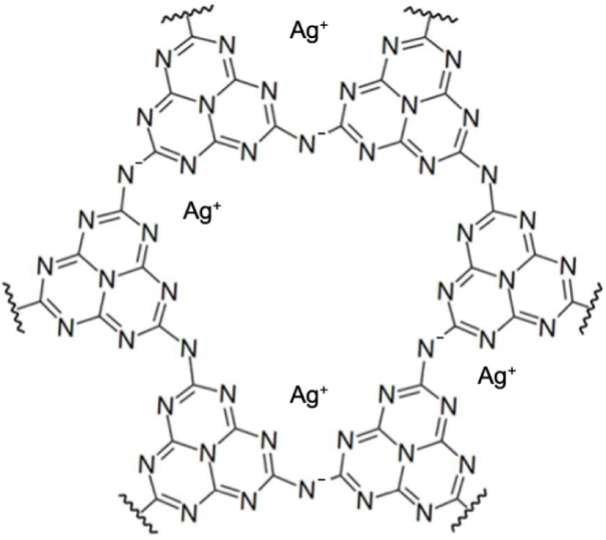
Molecular structure of Ag-poly­(heptazine imide) (Ag-PHI). The number of silver ions in pores of Ag-PHI was not experimentally determined.

## 2 Experimental Section

### 2.1 Preparation of K-PHI

For the synthesis of melon, the precursor of K-PHI, the quartz test tube/quartz tube used for calcination was first heated at 700 °C for 45 min in a tube furnace (JTEKT Thermo Systems Co., Ltd., KTF035N1). Melamine (3.0 g, purity: 99.0%, Wako Pure Chemicals Co., Ltd., 139-00945) was then placed in the quartz test tube, covered with an aluminum foil with a pinhole of ∼0.6 mm diameter in the center, and fixed with a tungsten wire. It was then placed in a quartz tube and synthesized under a nitrogen atmosphere (purity: 99.99995%). Synthesis was performed by heating to 550 °C at a rate of 1 °C min^–1^, holding at 550 °C for 5 h, and then cooling to room temperature at 2 °C min^–1^. For the synthesis of K-PHI, quartz test tubes and quartz tubes used for calcination were first heated at 700 °C for 45 min. Then, 0.3 g of the synthesized melon and 0.15 g of KSCN (purity: 98.0%, Wako Pure Chemicals Co. 164-04555) were mixed and placed in a calcination boat. They were covered with an aluminum foil in a quartz test tube and fixed with a tungsten wire. The synthesis was performed under a nitrogen atmosphere (purity: 99.99995%). The boat was rapidly heated to 400 °C at a heating rate of 30 °C min^–1^ and then held at 400 °C for 1 h. After the first heating step, the mixture was thermally treated again by heating to 500 °C at a heating rate of 30 °C min^–1^, held at 500 °C for 30 min, and then cooled to room temperature at a cooling rate of 2 °C min^–1^. The product was washed with acetone and water and separated by centrifugation. Finally, the sample was dried in a desiccator to obtain K-PHI as a yellow powder.

In order to stably induce photochromism in K-PHI, triethanolamine (TEOA: purity: 98.0%; Sigma-Aldrich) was used as an electron donor.

### 2.2 Preparation of H-PHI

Diluted sulfuric acid (0.05 vol %) was prepared from sulfuric acid (purity: 96–98%, Fujifilm Wako Pure Chemicals Co.). K-PHI was dispersed in dilute sulfuric acid at a concentration of 7.5 mg/mL and stirred at 500 rpm for 30 min. The dispersion was then sonicated for 10 min. The product was washed multiple times with pure water and dried in a desiccator.

### 2.3 Preparation of Ag-PHI

A silver nitrate solution (1.77 mol/L) was prepared by adding pure water to silver nitrate (purity: 99.8%, Fujifilm Wako Pure Chemicals Co.). H-PHI was dispersed in the silver nitrate solution at a concentration of 150 mg/mL and stirred at 500 rpm for 5 days. After that, the product was washed several times with pure water and dried in a desiccator. The samples were stored in aluminum foil to shield them from light. The structure and chemical state of the obtained samples were investigated by X-ray diffraction (XRD) and Fourier transform infrared spectroscopy (FTIR), and confirmed to be Ag-PHI (Figure S1).

### 2.4 Characterization

Ultraviolet (UV)-visible measurements (JASCO Corporation, V-670 equipped with an integrating sphere) were performed by placing the sample between quartz glass plates and measuring the reflectance of light. The osmium-coated samples were used for scanning electron microscopy (SEM; FE-SEM SUPRA40; Carl Zeiss). The osmium coating was performed with a coater (Neoc-Pro; MEIWAFOSIS, Ltd.), using osmium (VIII) oxide (purity: 99.8%; FUJIFILM Wako Pure Chem. Co., Ltd.; 157-00404). The thickness of the osmium coating was 5 nm.

Electric current measurements of the samples were performed using a source-measure unit (6487 J, Keithley) and a DC power source (R6144, Advantest).

## 3 Results and Discussion

Figure 2 shows the UV–vis spectra of Ag-PHI before and after irradiation with white light. Here, “Ag-PHI black” represents Ag-PHI that turned black after irradiation with white light. “K-PHI blue” represents K-PHI that turned blue when irradiated with white light.^33–35^ K-PHI blue is a powder sample made by mixing 20 mg of K-PHI powder with 30 mL of TEOA. Ag-PHI shows almost no absorbance in the wavelength range longer than 450 nm, whereas Ag-PHI black shows broad absorption with a low-intensity peak at approximately 600 nm, indicating that it absorbs light over a wide wavelength range in the visible region.

**Figure 2:**
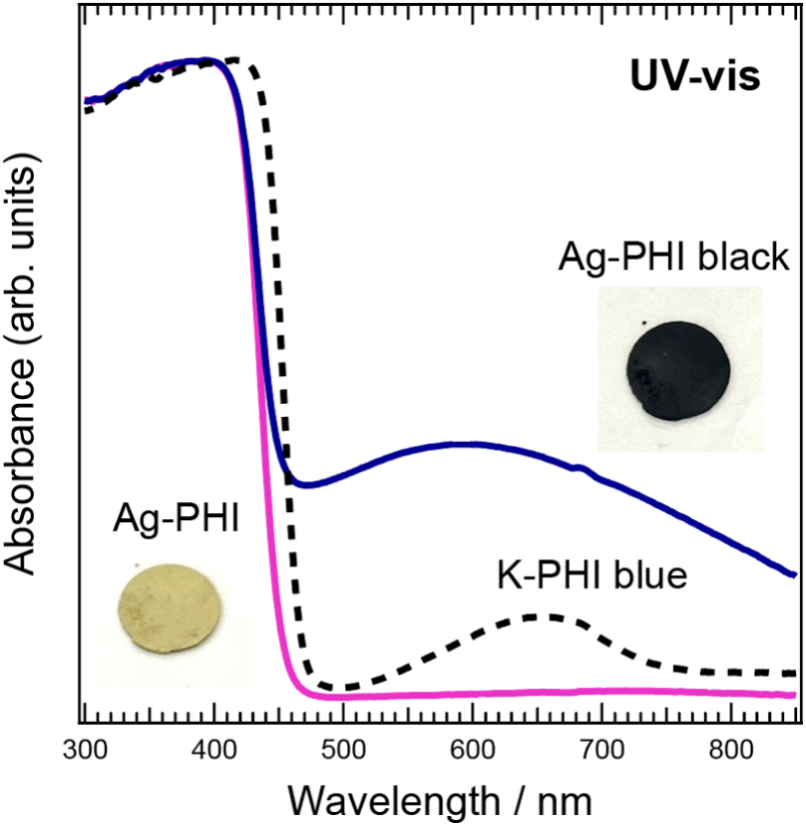
UV–vis spectra of powder samples of Ag-PHI, Ag-PHI black, and K-PHI blue. The spectrum of white-light source is shown in Figure S2. Horizontal axis: wavelength; vertical axis: absorbance. Photographs show pellets of Ag-PHI and Ag-PHI black after 30 min of photoirradiation of Ag-PHI. Diameter of pellets: 1 cm.

As shown in the SEM images (Figure 3a,b), the surface morphologies of Ag-PHI and Ag-PHI black were specific to layered materials, with sparsely distributed particles on the surfaces. The size distribution of the particles on the surface of Ag-PHI was mainly in the range of 10–20 nm, whereas that of Ag-PHI black was mostly in the range of 20–40 nm (Figure 3c,d). The particles on the surface of Ag-PHI black were larger than those on Ag-PHI because Ag^+^ was released from the PHI structure by photoirradiation, and the Ag particles aggregated to form Ag nanoparticles (Ag-NPs). White-light irradiation of K-PHI induces the desorption of K^+^ from the PHI structure due to changes in the charge distribution.^34^ Therefore, a similar change is expected for Ag-PHI. This hypothesis was confirmed by the observation of the (111) diffraction peak of the Ag crystal in the X-ray diffraction (XRD) spectrum of Ag-PHI black (Figure S3). This diffraction peak was not observed for Ag-PHI. The SEM image in Figure 3a shows Ag-NPs on the surface of Ag-PHI. Ag-PHI is only exposed to low-intensity indoor light during the experiments, such as the SEM observation. Therefore, it is plausible that these Ag-NPs precipitated in response to indoor light. This hypothesis was also confirmed by X-ray photoemission spectroscopy (XPS) measurements of Ag-PHI: the Ag 3d XPS spectrum shown in Figure S4 is well explained by the contribution of Ag^+^ bound to the PHI framework and Ag^0^ of Ag-NPs, indicating that Ag-PHI also contains small amounts of Ag-NPs. FTIR of Ag-PHI black (Figure S5) showed absorption due to stretching vibrations indicating that Ag^+^ is bound to the PHI framework. This result indicates that in Ag-PHI black, not all Ag^+^ is reduced to Ag-NP, but some Ag^+^ remains bound to the PHI framework.

**Figure 3:**
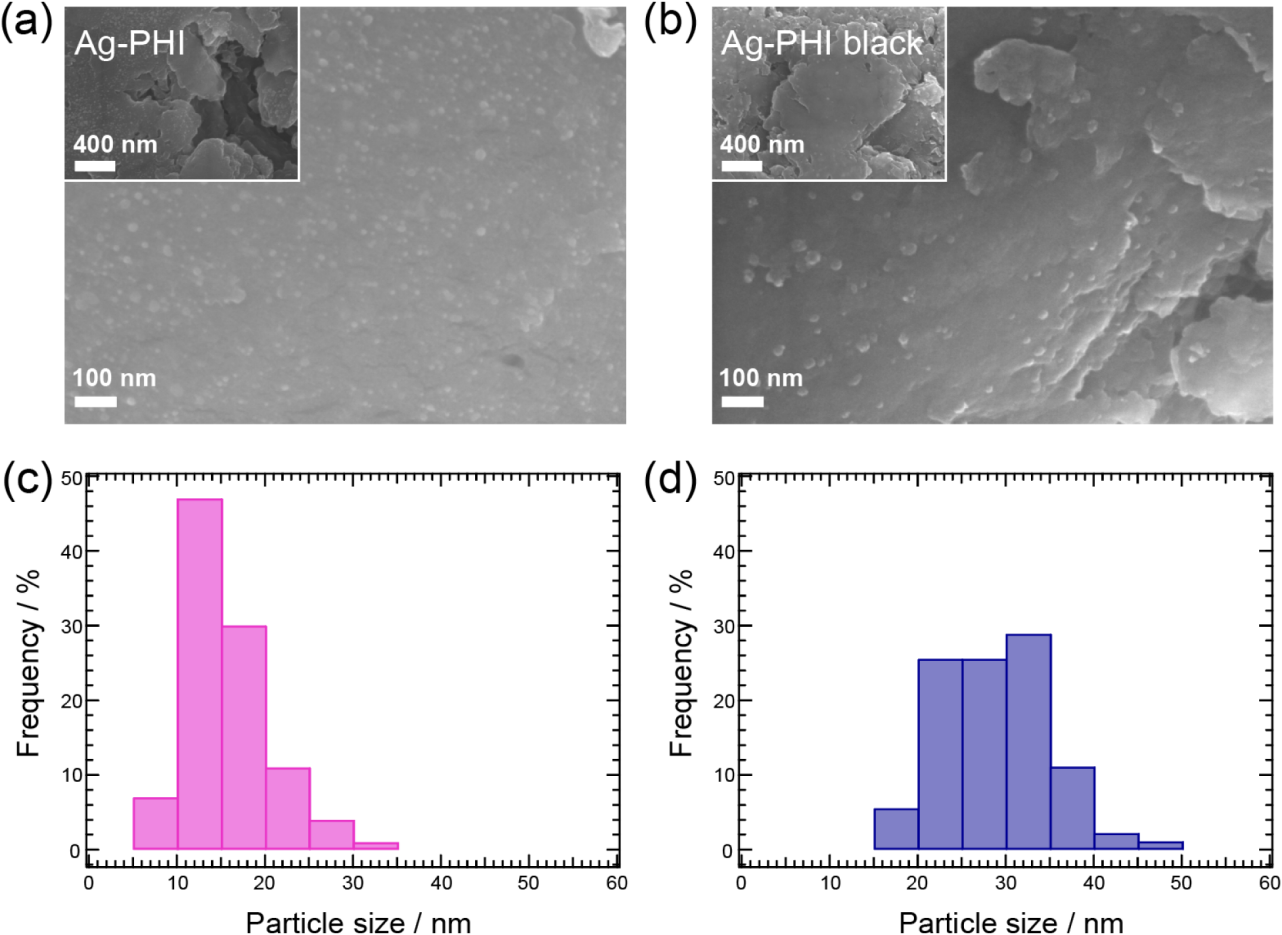
SEM images of Ag-PHI (a) and Ag-PHI black (b). Particle size distributions of Ag particles on the surface of Ag-PHI (c) and Ag-PHI black (d). The horizontal axis indicates the particle size, and the vertical axis indicates the percentage of the number of particles in each size interval out of the total number of particles. The particle size distribution was obtained by measuring the diameter of 100 particles selected at random in the SEM image.

Here, we again consider the broad band around 600 nm that appears in the absorption spectrum of Ag-PHI black. As mentioned, white-light irradiation of PHI containing alkali metal ions, such as K-PHI, leads to desorption of the ions from the PHI structure, and a new absorption band appears at approximately 670 nm,^33–35^ accompanied by a color change from yellow to blue. For comparison, the UV–vis spectrum of K-PHI that turned blue (K-PHI blue) after white-light irradiation is shown in Figure 2. This photochromism is caused by a change in the electronic structure of PHI due to the desorption of metal ions; therefore, a similar phenomenon is expected in Ag-PHI. However, in the case of other PHIs, the broadened absorption wavelength range gradually reverts to its prelight-irradiation state after photoirradiation, as the metal ions are gradually reabsorbed into the PHI structure. In contrast, Ag-PHI that turned black upon photoirradiation did not return to its original state. This observation indicates that the Ag^+^ removed from the PHI structure was not reabsorbed into the PHI structure. Ag^+^ released from the PHI structure by white-light irradiation is reduced by the photogenerated electrons in Ag-PHI, and Ag-NPs are deposited. Once the Ag-NPs precipitated, Ag^+^ was not reabsorbed into the PHI structure.

Ag-NPs absorb light in the visible region due to surface plasmon resonance (SPR) and exhibit vivid colors depending on their size and shape.^36,37^ However, the color of the dispersion changes to black as the concentration of the Ag-NPs increases^38^ due to aggregation of the Ag-NPs at high concentrations. This leads to broadening of the absorption peak, and another broad absorption band (called a secondary peak) appears on the long-wavelength side.^39^ The absorption band in the UV–vis spectrum of Ag-PHI black is much broader than the 670 nm peak observed for K-PHI blue (see Figure 2). Therefore, the absorption band of Ag-PHI black at wavelengths longer than 450 nm is attributed to aggregation of the Ag-NPs, in addition to the absorption peak at approximately 670 nm caused by the desorption of Ag^+^ from the PHI structure.

Figure 4 shows the time variation of the Ag-PHI current with the application of a voltage of 2 V. The electrical properties of Ag-PHI were measured by sandwiching a pellet-shaped Ag-PHI powder sample between an indium tin oxide (ITO) electrode (anode) and a Cu electrode (cathode). In Figure 4, the time period during which the sample is irradiated with white light is shown in the white area labeled “ON,” and the dark state is shown in the gray area labeled “OFF.” The conductivity of Ag-PHI decreased over time during photoirradiation (Figure 4a), indicating that Ag-PHI exhibited NPC. Also, as indicated by the double-headed arrow in the figure, the current differed before the first and second photoirradiation processes. To investigate the NPC of Ag-PHI in detail, the graph in Figure 4a was expanded to show the range from 0 to 3500 s (Figure 4b). The temporal change in the Ag-PHI current can be divided into four regions (Figure 4b), in which the current: (i) increases rapidly immediately after the start of photoirradiation; (ii) gradually decreases during photoirradiation; (iii) decreases rapidly immediately after the start of photoirradiation; and (iv) gradually increases during photoirradiation. Notably, as discussed above, Ag-PHI changed to Ag-PHI black after the first irradiation. In other words, when the current was measured (Figure 4a), Ag-PHI immediately changed to Ag-PHI black after the first photoirradiation. Therefore, the sample could be considered Ag-PHI black immediately after the first photoirradiation. On this basis, the mechanism of the NPC in Ag-PHI is discussed below.

**Figure 4:**
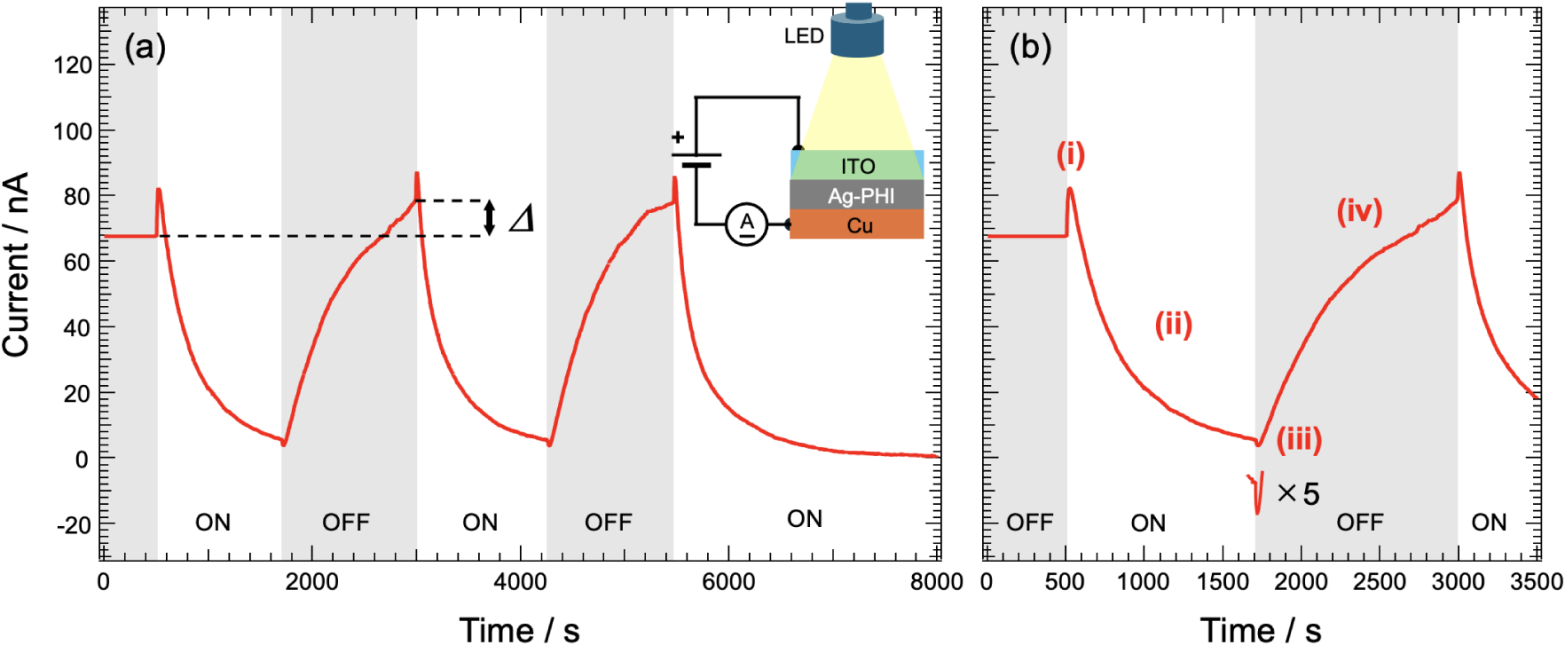
(a) Temporal evolution of electrical current upon applying a constant voltage of 2 V to Ag-PHI. Horizontal axis: time; vertical axis: electrical current. In the time range indicated by the white band, Ag-PHI was irradiated with white light, and in the range shaded in gray, there was no irradiation with white light. The double-headed arrows indicate the difference in the current before the first and second photoirradiation processes. (b) Enlarged graph of 0–3500 s range in Figure a; current at 8000 s was set to 0 nA; schematic of the circuit used in the measurements.

Before discussing the mechanisms of NPC in Ag-PHI, some characteristics of Ag-PHI are first reviewed. First, M-PHI is a semiconductor with n-type characteristics (here, M represents a monovalent metal ion or proton). Many M-PHI compounds containing alkali metals are ionic conductors; however, they also exhibit semiconductor characteristics because of the π-conjugated system in the PHI structure.^30,40–43^ As discussed above, it should be noted that Ag-PHI is a semiconductor, but not an ion conductor. Second, M-PHI is a photoconductor that exhibits PPC. Therefore, it is reasonable to assume that Ag-PHI also exhibits PPC because of charge separation of the excitons generated by light absorption (Figure 5a-1), given that the semiconductor properties are derived from the PHI structure regardless of the type of metal contained in PHI. When Ag-PHI is irradiated with white light, Ag^+^ is released from the PHI structure and reduced by excited electrons in the conduction band (CB), forming Ag-NPs (Figure 5a-2). The holes remaining in the valence band (VB) oxidize the water molecules in Ag-PHI. M-PHI absorbs water molecules from the pores of the PHI structure. Electron spin resonance (ESR) experiments (Figure S7) confirmed that compared to low-humidity environments, the number of unpaired electrons generated in Ag-PHI by photoirradiation is lower in high-humidity environments. Thus, it can be assumed that the holes accumulated in Ag-PHI were consumed by the oxidation of water molecules. Third, M-PHI releases metal ions from the PHI structure upon photoirradiation. It is known that metal ions are removed and the remaining negatively charged nitrogen atoms are protonated to form H-PHI. Thus, it is inferred that Ag-PHI black produced by white-light irradiation of Ag-PHI has a structure in which H-PHI and the Ag-NPs are embedded in Ag-PHI (see lower part of Figure 5b).

**Figure 5:**
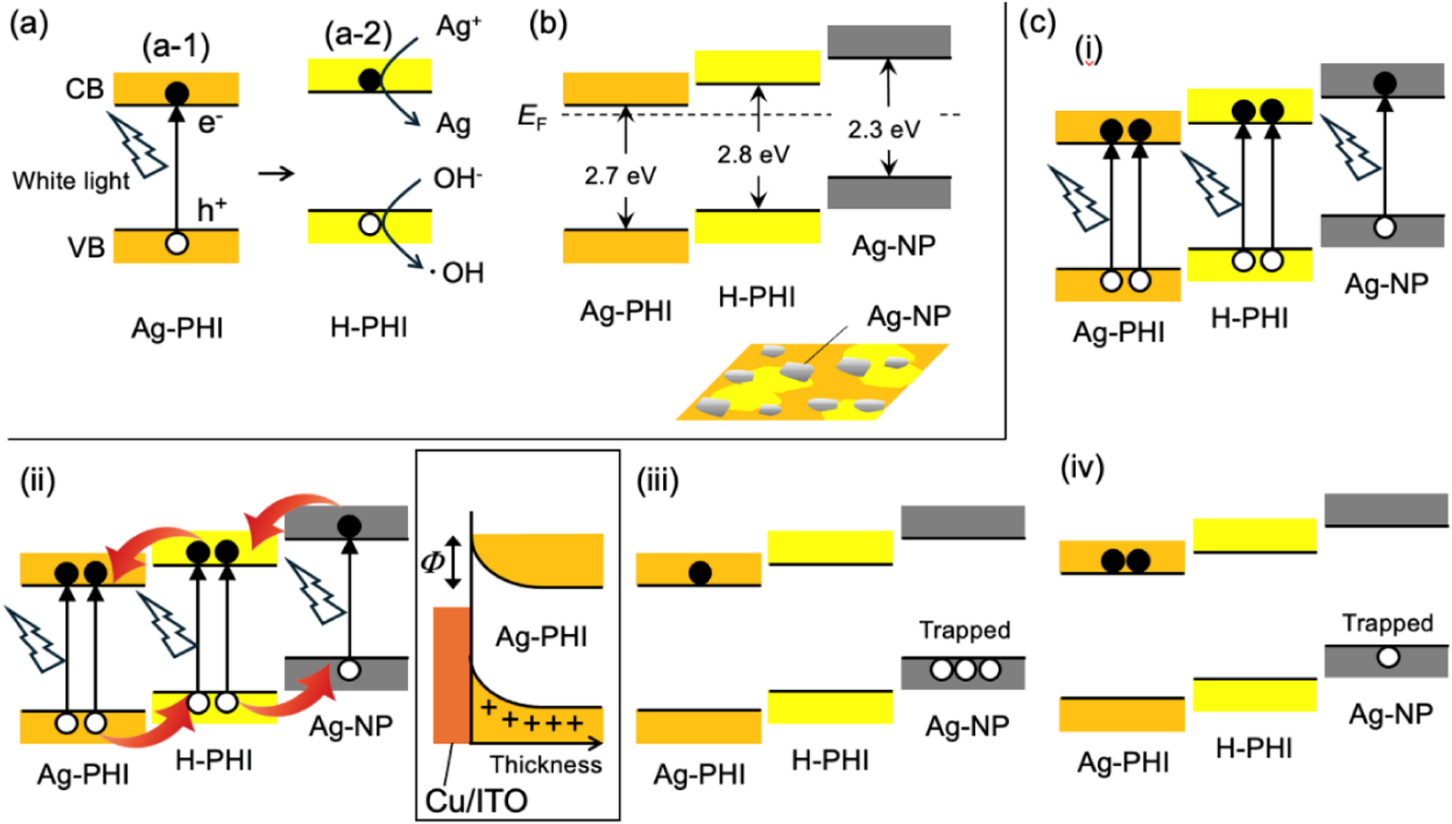
(a) Reactions upon irradiating Ag-PHI with white light. (b) Relationship between energy of valence band (VB) and conduction band (CB) of Ag-PHI, H-PHI, and Ag-NP. The dotted line indicates the Fermi level (*E*_F_). The energies of the VB of Ag-PHI and Ag-NP were determined from the ultraviolet photoemission spectroscopy (UPS) spectrum of Ag-PHI shown in Figure S6. The illustration in Figure (b) is a schematic of the surface of Ag-PHI black. The orange and yellow regions represent Ag-PHI and H-PHI, respectively. (c) Schematic energy diagrams of Ag-PHI black at each stage (i–iv) of the time development of the current are shown in Figure 4b. The diagram enclosed in a square is the energy diagram of the electrode interface at stage (iii).

The energy diagrams of Ag-PHI, H-PHI (generated by irradiating Ag-PHI with white light), and the Ag-NPs are shown in Figure 5b. The upper edges of the VB of the Ag-PHI and Ag particles were determined using UPS (Figure S6). The energy gaps of Ag-PHI and H-PHI were estimated from the absorption edges of the respective UV–vis spectra (Figure S8). The energy gap of Ag particles strongly depends on their size. Therefore, the literature value of the energy gap of Ag-particles with a size close to that observed in the SEM image in Figure 3b was used herein.^44^ Previous studies have shown that the CB of PHI after protonation is located at a higher energy level than that before protonation.^29^ Therefore, the CB of H-PHI was plotted at a higher energy level than that of Ag-PHI. These diagrams are meaningful for the current discussion, but are only schematic diagrams; therefore, they need to be verified experimentally in the future.

Based on the energy diagram in Figure 5b, the time evolution of the Ag-PHI current shown in Figure 4b is discussed. First, when Ag-PHI was irradiated with white light, Ag^+^ was immediately desorbed from Ag-PHI to form H-PHI and Ag-NPs. This can be seen from the fact that Ag-PHI immediately turned black when irradiated with white light. Therefore, in state (i) of Figure 4b, Ag-PHI, H-PHI, and the Ag-NPs exhibited PPC upon light absorption. This caused a spike-like increase in the current immediately after white-light irradiation. White-light irradiation then produces a slow decrease in the current, or NPC, in the subsequent stage (ii). Judging from the current in the 5400–8000 s range in Figure 4a, the gradual decrease in the current caused by NPC occurred over a period of 1500 s or more, and after photoirradiation for approximately 2000 s, the current did not decrease any further. This decrease in current can be expressed as a superposition of two exponential functions of time, and the results of the fitting analysis (Figure S9a) showed that the time constant was 383.1 ± 1.3 s. The situation in stage (ii) is shown in diagram (ii) in Figure 5c. The excitons generated in the Ag-PHI, H-PHI, and Ag-NPs by photoirradiation were separated into electrons and holes. Because the energies of the CB and VB are highest in the Ag-NPs and lowest in Ag-PHI, electrons are transferred to Ag-PHI, and holes are collected by the Ag-NPs. The electrons flow through Ag-PHI, and the current flows. However, as shown at the bottom of Figure 5b, the Ag-NPs are surrounded by Ag-PHI and H-PHI; thus, holes are trapped within the Ag-NPs. NPC is caused by a decrease in the number of carriers in Ag-PHI and H-PHI, owing to the trapping of holes in the Ag-NPs. However, it is not possible to fully understand the NPC exhibited by Ag-PHI using hole trapping by the Ag-NPs alone because the number of holes trapped in Ag-NPs is finite, and when the number of trapped holes reaches saturation, the current should recover. However, as mentioned above, the current does not recover but remains low even if photoirradiation is continued. Furthermore, since Ag-PHI is expected to have n-type characteristics, the main carrier is an electron. Therefore, the trapping of holes by Ag-NPs alone cannot explain the result that almost no current flows with continuous light irradiation, as shown in Figure 4. This phenomenon can be explained by the distribution of the positively charged Ag-NPs in Ag-PHI black. By solving the Poisson equation with a spatially uniform distribution of positive charge density, the potential energy decreases as the charge density moves away from the electrode interface, causing downward band-bending. This band-bending creates a potential barrier at the electrode interface, making it difficult to inject electrons into or extract them from Ag-PHI black. A conceptual diagram of this scenario is shown in box (ii) of Figure 5c. This barrier continued to reduce the electron current of Ag-PHI black as long as the holes trapped in the Ag-NPs were not released. In (iii), when photoirradiation was discontinued, the generation of photocarriers stopped; therefore, the number of carriers in Ag-PHI black decreased, causing the current to decrease with a dip-like profile. In stage (iv), the holes trapped in the Ag-NPs were gradually released by thermal excitation; therefore, the barrier at the interface was slowly eliminated, and the electron current gradually recovered. This increase in current can be expressed as an exponential function of time, and the result of the fitting analysis (Figure S9b) showed that the time constant was 516.4 ± 1.8 s. In stage (iv), current recovery is very slow. Indeed, when the current–voltage characteristic was measured immediately after white-light irradiation to produce Ag-PHI black, the electrical conductivity of Ag-PHI black remained very low compared to that of Ag-PHI (Figure S10). When Ag-PHI black was again irradiated with white light, steps (i) to (iv) were repeated. On the other hand, as indicated by *Δ* in Figure 4a, the current generated by Ag-PHI black is greater than that generated by Ag-PHI before the first photoirradiation step, and the current generated by Ag-PHI black is greater than that generated by Ag-PHI before the second and subsequent photoirradiation steps. In other words, Ag-PHI black exhibits higher electrical conductivity than Ag-PHI. A similar phenomenon has been observed in the NPC of other substances, and a previous study reported that the electrical conductivity of a sample with loaded metal particles was improved compared to that of the sample before loading.^6^ The NPC mechanism proposed herein for Ag-PHI is only a conjecture, and experimental verification is required in the future. However, it should be emphasized that this is a new perspective for elucidating the NPC mechanism, as it considers the effect of the barrier formed at the interface, rather than the previously reported NPC that occurs directly by carrier trapping by the loaded metal particles.^1–3,45^

## 4 Conclusion

The present study investigated the mechanism of NPC in Ag-PHI. Examination of the structure before and after white-light irradiation demonstrated that Ag-PHI black, generated in response to photoirradiation, comprised a mixture of Ag-PHI, H-PHI, and Ag-NP. Examination of the photoresponse current revealed that Ag-PHI exhibits NPC. Many studies have reported that NPC is caused by the deposition of metal particles on semiconductors, suggesting that carrier trapping due to trap levels caused by metal particles is the origin of NPC. Ag-PHI black contains many Ag-NPs that were deposited upon photoirradiation; therefore, a similar mechanism is expected, but this mechanism cannot fully explain the NPC exhibited by Ag-PHI black. Therefore, a new mechanism is proposed, in which the potential barrier formed at the electrode interface with Ag-PHI black can also cause NPC. At present, PHI species containing various metals are being actively studied, but this report is the first discovery of a PHI exhibiting NPC, and is one of the few reports on organic semiconductors exhibiting NPC. Therefore, the development of new applications of NPC that cannot be achieved using conventional inorganic semiconductors is anticipated. In addition, the NPC exhibited by Ag-PHI black occurred over 1500 s. This is not suitable for application in photodetectors but may be useful for applications such as synaptic devices.

## Supplementary Material



## Notes

XRD patterns and FTIR spectra of Ag-PHI and K-PHI, spectrum of the white-light LED used to measure the electrical characteristics, XRD patterns of Ag-PHI and Ag-PHI black, Ag 3d XPS spectrum of Ag-PHI, FTIR spectra of Ag-PHI and Ag-PHI black, UPS spectrum of Ag-PHI black, ESR signals of Ag-PHI before and after white light irradiation at different relative humidities, UV–vis spectra of Ag-PHI and H-PHI, fitting analysis of temporal evolution of electrical current of Ag-PHI, and current–voltage characteristics of Ag-PHI and Ag-PHI black (PDF)
