# Supplementary material for: Negative Photoconductivity of Ag-Poly(heptazine imide)
Source: ACS Omega. 2025 Jul 8;10(28):30424–31. doi: 10.1021/acsomega.5c02134 (PMC12290614; doi:10.1021/acsomega.5c02134)
Supplement: Supplementary file 1 [file ao5c02134_si_001.pdf]

Supporting information

Negative photoconductivity of Ag-poly(heptazine imide)

Chihiro Miyazaki[1], *Momoka Isobe*[1,*], *Yunosuke Takezawa*[1], *Ayane Nakamura*[1], *Mai Hattori*[1], *Ryosuke Ohnuki*[1], *Shinya Yoshioka*[1], *Kaname Kanai*[1]

[1]*Department of Physics and Astronomy, Faculty of Science and Technology, Tokyo University of Science, 2641 Yamazaki, Noda, Chiba 278-8510, Japan*

[*]Corresponding author: 6223504@ed.tus.ac.jp

Experimental: Powder X-ray diffraction (XRD) patterns were recorded using a diffractometer (Rigaku, Ultima IV) with a Cu-Kα radiation source. XPS (JPS-9030/JEOL Ltd.) measurements were performed using Al Kα radiation (λ = 1486.6 eV) as the excitation source. The XPS profiles were analyzed using Voigt functions with XPSPEAK41 software (written by Raymund W. M. Kwok). Fourier transform infrared (FTIR) spectra of the samples embedded in KBr pellets were acquired using a spectrometer (JASCO Corporation, FT/IR-6100). Ultraviolet photoemission spectroscopy (UPS) measurements were performed using SES200 (SCIENTA) as analyzer and He Iα ($h\nu$=21.22 eV) as light source. Electron spin resonance (ESR) measurements were performed with EMXnano (Bruker). ESR measurements were performed on a powder sample (10 mg) placed in a quartz ESR tube with outer diameter 5 mm (Agri, ST-X-5-3).

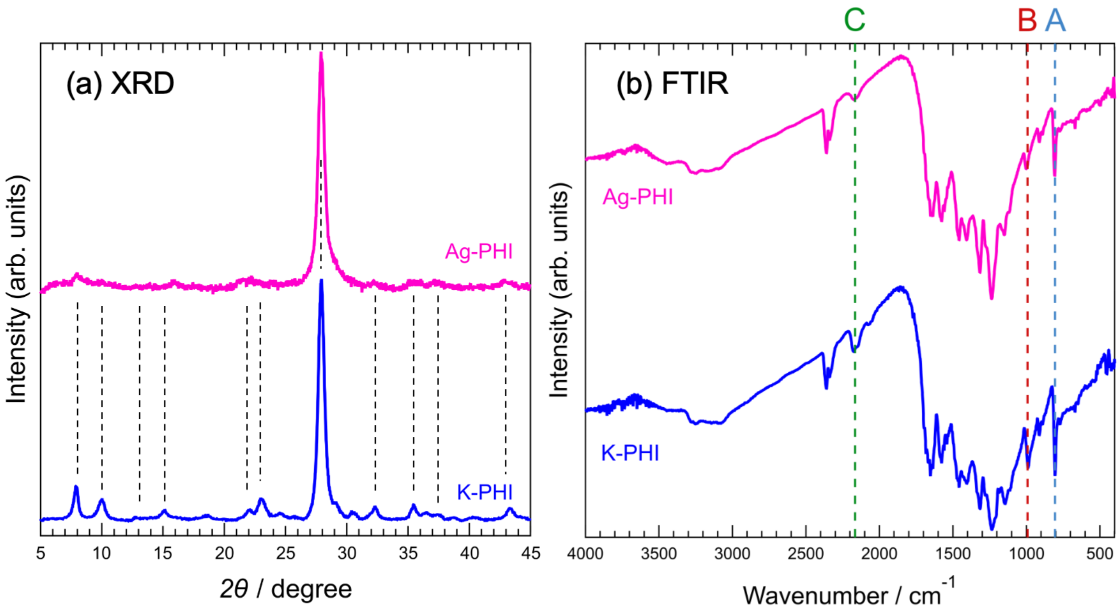

Figure S1. (a) XRD patterns of Ag-PHI and K-PHI. The horizontal axis is the diffraction angle 2θ and the vertical axis is the diffraction intensity. (b) FTIR spectra of Ag-PHI and K-PHI.

Figure S1(a) shows the XRD patterns of the Ag-PHI and K-PHI powders synthesized in this study; although the intensities of diffraction peaks of Ag-PHI are lower than those of K-PHI, the diffraction patterns are basically the same. In particular, the peaks at $2\theta = 7° \sim 10°$ and $2\theta \simeq 28°$ are characteristic of PHI, suggesting that Ag-PHI has a similar structure to that of K-PHI. The former peaks are diffraction peaks derived from the in-layer period of the PHI structure, while the latter peak is derived from the period between PHI layers. On the other hand, the peak intensity observed at $2\theta = 10°$ is lower than that observed at $2\theta = 8°$ in Ag-PHI. This may be due to the irregular slip between PHI layers.[1] In addition, the relative ratio of diffraction intensities within the layer may change depending on the arrangement of water molecules present in the pores of the PHI structure. The FTIR spectra of Ag-PHI and K-PHI in Figure S1(b) are very similar

to each other, indicating that they have the same structure. The sharp peak at 810 cm^{-1}, indicated by A in the figure, is due to the angular vibration of the tri-s-triazine (heptazine) unit. In addition, the broad wavenumber band from 1100 to 1700 cm^{-1} is called the fingerprint region, and it shows absorption due to the C-N stretching vibration and N-H bending vibration of the heptazine ring. Since the spectral features in the fingerprint region of Ag-PHI is very similar to those of K-PHI, it can be seen that Ag-PHI also has a PHI structure. Furthermore, the peak at about 1000 cm^{-1} indicated by B in the figure is assigned to the absorption due to the stretching vibration of the metal ion-NC_2 bond, and is also observed in Ag-PHI. This fact indicates that the Ag^+ is bound to the NC_2 site of the PHI structure in Ag-PHI.

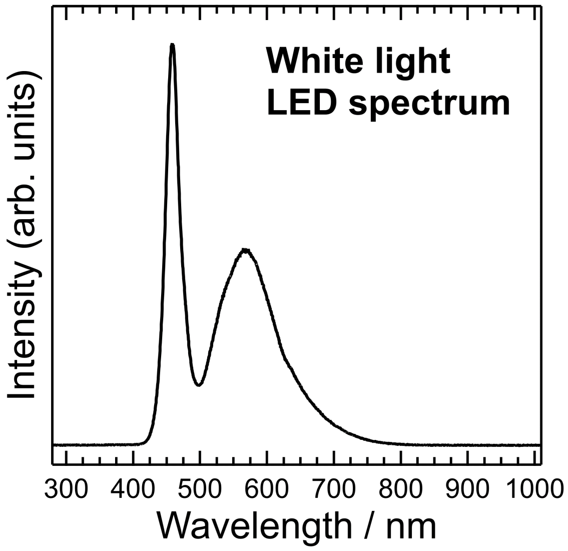

Figure S2. Spectrum of the white-light LED used to measure the electrical characteristics shown in Figures 2 and 4.

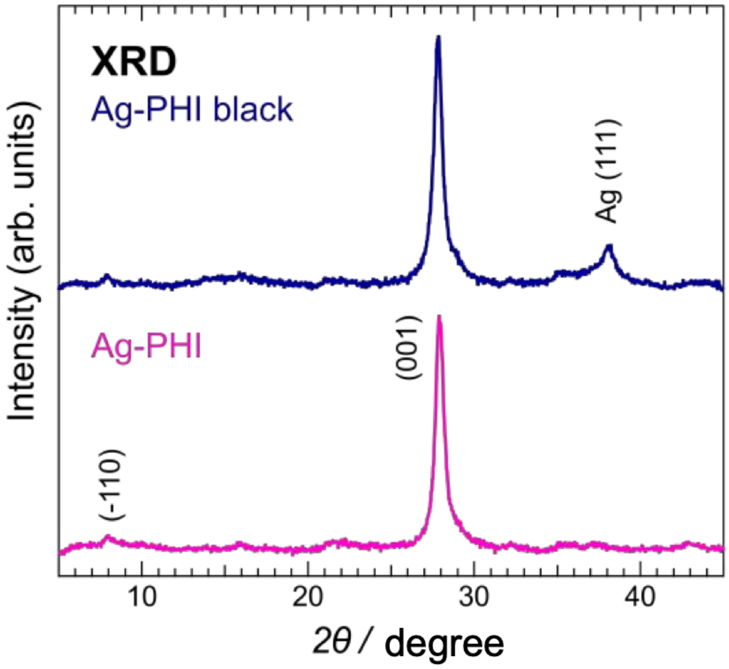

Figure S3. XRD patterns of Ag-PHI and Ag-PHI black.

Figure S3 shows the XRD patterns of Ag-PHI and Ag-PHI black. The XRD pattern of Ag-PHI black shows diffraction peaks at 2θ = 7.8°, 27.8°, and 38.3°. The peaks at 2θ = 7.8° and 27.8° also appear in Ag-PHI. The peak at 2θ = 7.8° corresponds to the in-plane periodic structure, and the peak at 2θ = 27.8° corresponds to the stacking direction periodic structure of PHI structure.[2,3] The fact that these peaks also exist in the Ag-PHI black indicates that the PHI structure is maintained even after light irradiation. In addition, the peak at 2θ = 38.3° corresponds to the XRD pattern of the Ag particles shown.[3] On the other hand, no peaks corresponding to the Ag_2O XRD pattern were observed. From the above, it was confirmed that Ag-PHI black maintains the PHI structure and that Ag particles are precipitated, and silver oxide has not been generated.

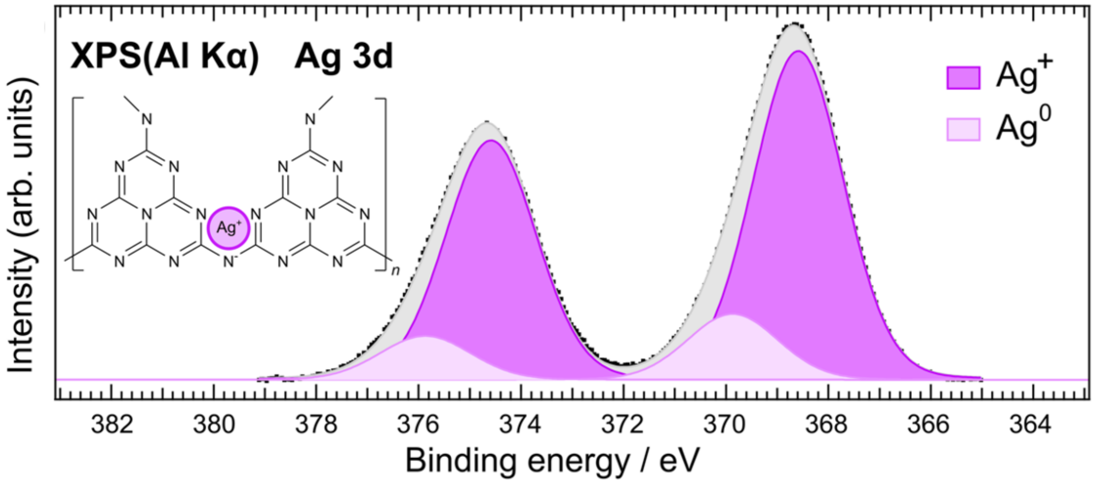

Figure S4. Ag 3d XPS spectrum of Ag-PHI.

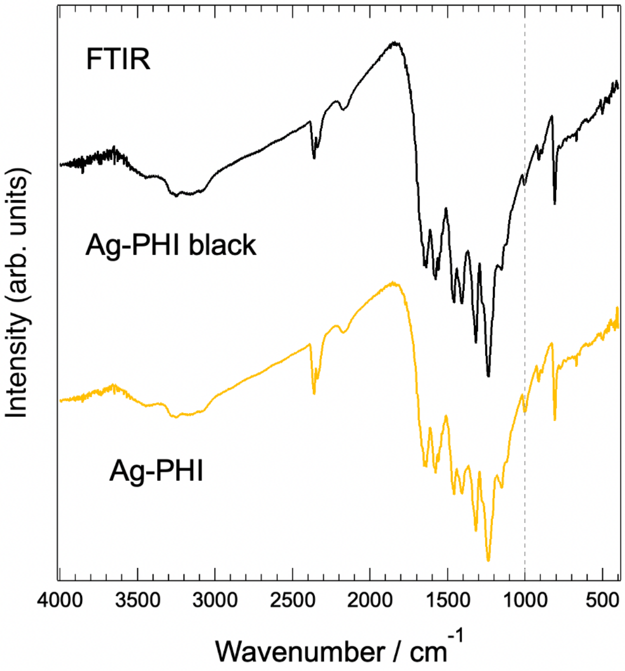

Figure S5. FTIR spectra of Ag-PHI and Ag-PHI black.

The absorption peak at about 1000 cm^{-1} indicated by broken line in the figure is assigned to the absorption due to the stretching vibration of the Ag^{+}-NC_2 bond. Compared to Ag-PHI, the absorption intensity of this stretching vibration is lower in the

Ag-PHI black spectrum. This result indicates that Ag^+ has been desorbed from the PHI framework by white-light irradiation, but also shows that some Ag^+ remains bound to the PHI framework even in Ag-PHI black.

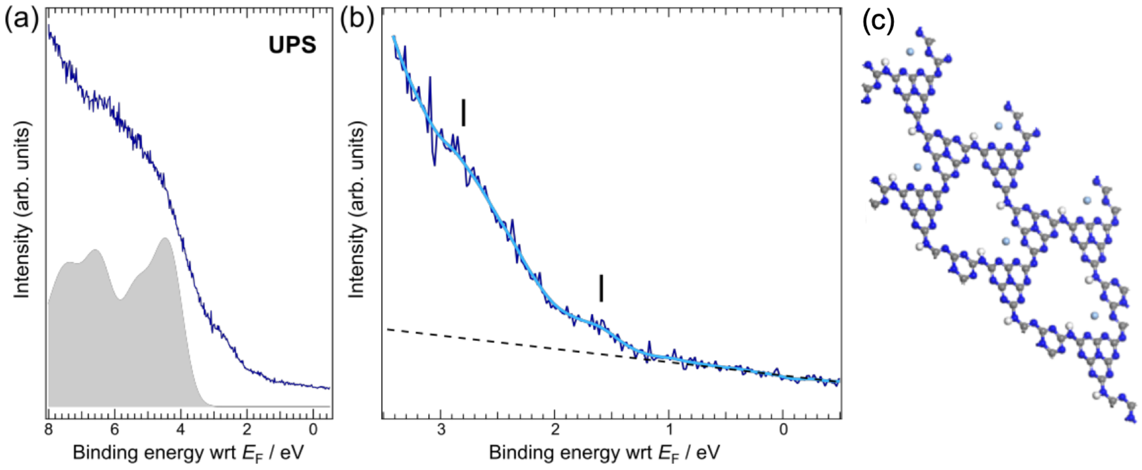

Figure S6. (a) UPS spectrum of Ag-PHI black. The horizontal axis is the binding energy relative to the Fermi level, and the vertical axis is the intensity of the photoelectron. The filled spectrum was obtained by convolving the calculated density of states for Ag-PHI with a Voigt function. The calculated spectrum is shifted to reproduce the structure of the valence band of the UPS spectrum. (b) An enlarged view of the range of binding energies from -0.5 eV to 3.5 eV in the graph in (a). The dotted line is the background, and the light blue line is the smoothed data. (c) The structure of Ag-PHI used in the simulation. The light blue spheres in the pores represent Ag^+.

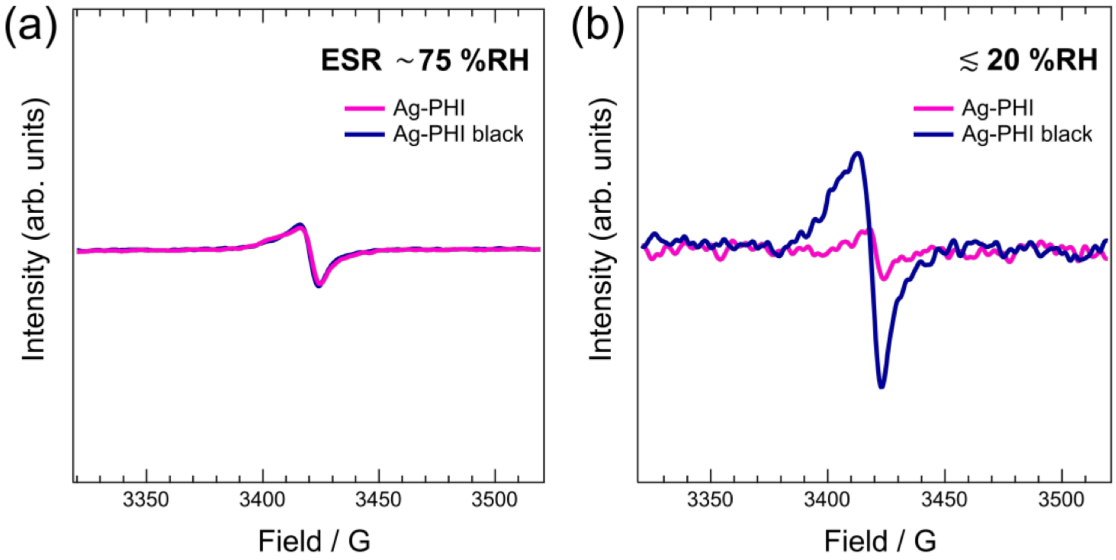

Figure S7. ESR signals of Ag-PHI before and after white light irradiation at different relative humidities (%RH). The horizontal axis is the applied magnetic field, and the vertical axis is the intensity of the ESR signal. (a) ESR signals of Ag-PHI and Ag-PHI black at ~75 %RH. (b) ESR signals of Ag-PHI and Ag-PHI black at ≲ 20 %RH.

As shown in Figure S7(a), the ESR signal intensity does not change between Ag-PHI and Ag-PHI black at 75%RH. This indicates that almost all of the holes generated in Ag-PHI are consumed in the oxidation of water molecules under this humidity condition. Therefore, further humidification is unlikely to promote NPC.

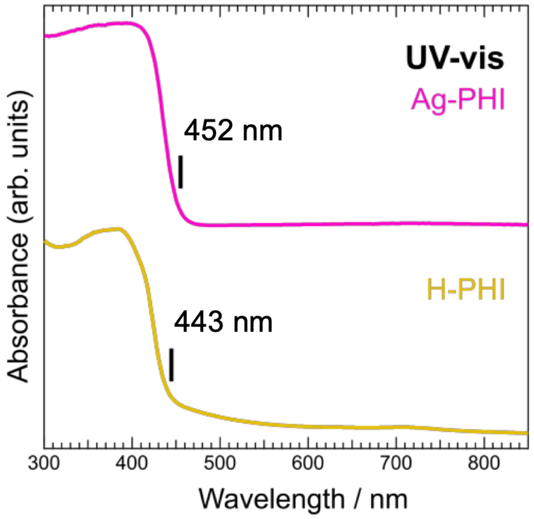

Figure S8. UV-vis spectra of Ag-PHI and H-PHI. The horizontal axis is wavelength and the vertical axis is absorbance. The black vertical lines in the figure indicate absorption edges.

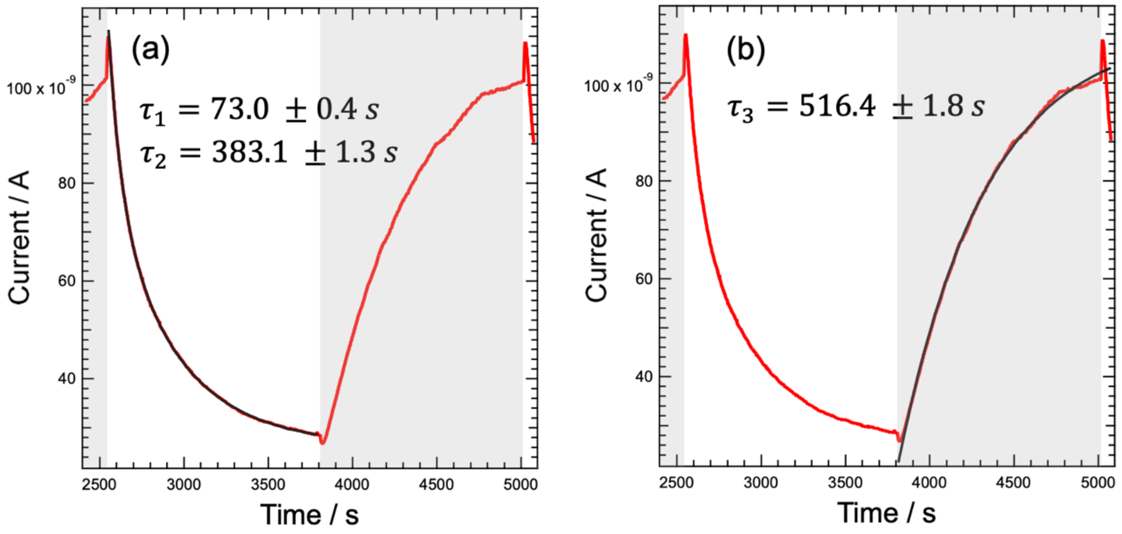

Figure S9. Temporal evolution of electrical current upon applying a constant voltage of 2 V to Ag-PHI. Horizontal axis: time; vertical axis: electrical current. In the time range indicated by the white band, Ag-PHI was irradiated with white-light, and in the range

shaded in gray, there was no irradiation with white light. The solid lines on the graph represent the results of the fitting analysis.

Figure S9 shows the results of fitting analysis of the electrical current plotted against time in Figure 4(b). Figure S9(a) shows the results of analyzing the decrease in current under light irradiation using equation (1), and Figure S9(b) shows the results of analyzing the increase in current after light irradiation was stopped using equation (2).

$$I(t) = I_0 + I_1 \mathrm{exp}\left\{-\frac{(t-t_0)}{\tau_1}\right\} + I_2 \mathrm{exp}\left\{-\frac{(t-t_0)}{\tau_2}\right\} \quad (1)$$

$$I(t) = I_3 + I_4 \mathrm{exp}\left\{-\frac{(t-t_1)}{\tau_3}\right\} \quad (1)$$

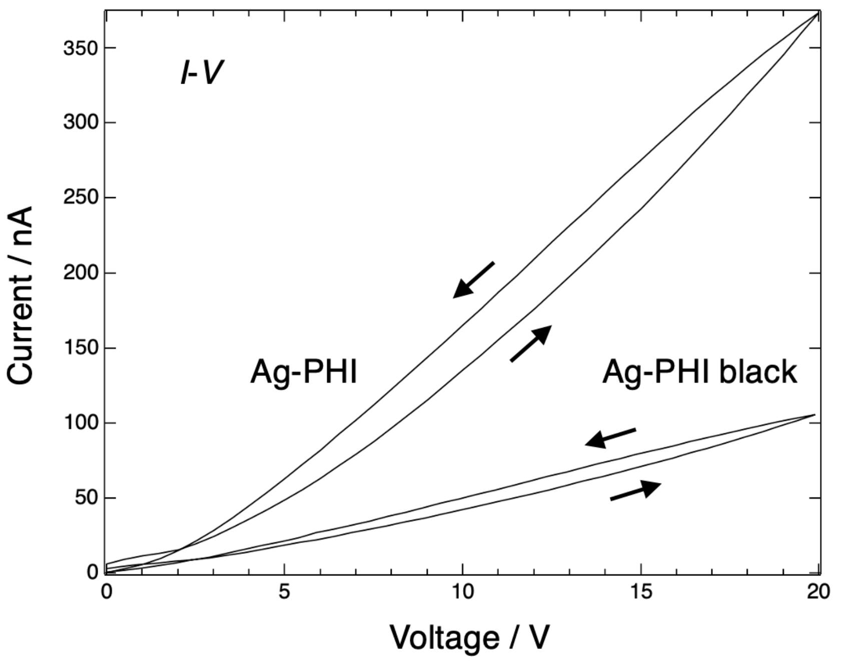

Figure S10. Current–voltage (*I–V*) characteristics of Ag-PHI and Ag-PHI black. Measurements were conducted using each pellet samples. The sample of Ag-PHI black was prepared by irradiating Ag-PHI with white-light for 5 min. Measurements were taken for 80 s immediately after stopping the white light irradiation. The schematic of the measurement circuit for a pellet sample was shown in Figure 4(a).

Figure S10 shows the *I–V* characteristics of Ag-PHI and Ag-PHI black. The electrical conductivity evaluated from the *I–V* characteristics in the range of 5 ~ 15 V is (8.56 ± 0.04) × 10^{-8} S/m and (2.34 ± 0.01) × 10^{-8} S/m for Ag-PHI and Ag-PHI black, respectively.

References

(1) Schlomberg, H.; Kröger, J.; Savasci, G.; Terban, M. W.; Bette, S.; Moudrakovski, I.; Duppel, V.; Podjaski, F.; Siegel, R.; Senker, J.; Dinnebier, R. E.; Ochsenfeld, C.; Lotsch, B. V., Structural Insights into Poly(Heptazine Imides): A Light-Storing Carbon Nitride Material for Dark Photocatalysis. Chem. Mater., **2019**, *31*, 7478－7486, DOI: 10.1021/acs.chemmater.9b02199

(2) Zihao, L.; Chenxi, L.; Zhu, S.; Lun, Z.; Tiantian, L.; Wenbing, W.; Zhengliang, Z.; Lina, X.; Lulu, S.; Lingling, F., K-Na Co-Doping in Crystalline Polymeric Carbon Nitride for Highly Improved Photocatalytic Hydrogen Evolution, Int J Hydrogen Energy., **2021**, *46* (52), 26318–26328, DOI: 10.1016/j.ijhydene.2021.05.138

(3) Savateev, A.; Pronkin, S.; Willinger, M. G.; Antonietti, M.; Dontsova, D., Towards Organic Zeolites and Inclusion Catalysts: Heptazine Imide Salts Can Exchange Metal Cations in the Solid State, Chem Asian J., **2017**, *12* (13), 1517–1522, DOI: 10.1002/asia.201700209

(4) Yang, H.; Ren, Y. yu; Wang, T.; Wang, C., Preparation and Antibacterial Activities of $Ag/Ag^{+}/Ag^{3+}$ Nanoparticle Composites Made by Pomegranate (Punica Granatum) Rind Extract, Results Phys., **2016**, *6*, 299–304, DOI: 10.1016/j.rinp.2016.05.012
